# *Acta Veterinaria Scandinavica* reviewer acknowledgement 2014

**DOI:** 10.1186/s13028-015-0152-9

**Published:** 2015-09-25

**Authors:** Jørgen S. Agerholm

**Affiliations:** Department of Large Animal Sciences, Faculty of Health and Medical Sciences, University of Copenhagen, Dyrlægevej 68, 1870 Frederiksberg C, Denmark

## Contributing reviewers

The *Acta Veterinaria Scandinavica* editorial team would like to thank the following colleagues who contributed to peer review for the journal in 2014.

**Albania**

Hal Herzog

**Argentina**

Rodolfo Luzbel de la Sota

**Australia**

Nicky Buller

Robert Kinobe

Mary-Louise McLaws

Nerissa Stander

**Austria**

Marc Drillich

Martina Flöck

**Belgium**

Ginette Deby-Dupont

Jeroen Dewulf

Jean-Claude Dujardin

Theo Niewold

Annemie Van Caelenberg

Heidi Wyns

**Brazil**

Helida Andrade

Ricardo Fujiwara

Guendalina Oliveira

**Bulgaria**

Milen Georgiev

**Canada**

Burim Ametaj

Wangxue Chen

Michael Cockram

Etienne Cote

Elaine Debien

Anli Gao

Jean-Pierre Lavoie

Jérôme Planté

Brandon Plattner

Bruce Rathgeber

Catherine Wagg

Scott Weese

**Denmark**

Klas Abelson

Jens Frederik Agger

Mohammad Al-Sabi

Jens Arnbjerg

Rikke Buhl

Henrik Callesen

Rikke Fast

Sandra Goericke-Pesch

Carsten Grøndahl

Luca Guardabassi

Christian Fink Hansen

Steffen Heegaard

Stine Jacobsen

Henrik Elvang Jensen

Tim K. Jensen

Sven Erik Jorsal

Nanna Luthersson

Liza Nielsen

Ole Lerberg Nielsen

Lisbeth Høier Olsen

Lene juul Pedersen

Christian Bressen Pipper

Erik Rattenborg

Tine Søland

Anne-Helene Tauson

Stig Milan Thamsborg

Tobias Wang

Nora Elisabeth Zois

**Estonia**

Pikka Jokelainen

Brian Lassen

Kerli Raaperi

Lea Tummeleht

Arvo Viltrop

**Ethiopia**

Zewdu Seyoum

**Finland**

Maria Fredriksson-Ahomaa

Laura Hanninen

Mari Heinonen

Sami Junnikkala

Outi Laitinen-Vapaavuori

Jere Linden

Tapani Lyytikäinen

Camilla Munsterhjelm

Antti Oksanen

Olli Peltoniemi

Merja Rantala

Ulla Rikula

Mirja Ruohoniemi

Seppo A. M. Saari

Antti Sukura

Pernilla Syrjä

Suvi Taponen

Pirkko Lucia Tuominen

Pekka Uimari

Anna Elisabet Valros

**France**

Francois Beaudeau

Gilles Charpigny

Sylvie Chastant-Maillard

Philippe Hennet

Eric Richard

Henri Seegers

Claudia Terlouw

Luca Zilberstein

**Germany**

Thomas Flegel

Klaus Henning

Korinna Huber

Stefanie Klenner

Arno Lindner

Burkhard Malorny

Ralf Mueller

**Greece**

Ilias Giannenas

**Iceland**

Sigridur Bjornsdottir

Ólafur R. Dýrmundsson

**India**

Praveen Malik

**Ireland**

John Mee

Keelin O’Driscoll

Cliona O’Farrelly

Luke O’Grady

**Israel**

Nahum Shpigel

**Italy**

Davide De Lorenzi

Nicola Decaro

Angela Di Cesare

Giulio Grandi

Vittorio Guberti

Luciana Mandrioli

Leonardo Nanni Costa

Erminio Trevisi

**Japan**

Keiichi Higuchi

Koichi Kadota

**Korea, South**

Jongki Cho

Han Sang Yoo

**Malaysia**

Mohd Effendy Abd Wahid

**The Netherlands**

Niklas Bergknut

Paul Dobbelaar

Birgitta Duim

Wouter Hendriks

Willie Loeffen

Paul Mandigers

Auke Schaefers-Okkens

J. A. Schrickx

Engeline van Duijkeren

Cornelis van Elk

**New Zealand**

Richard Laven

Mairi Stewart

**Norway**

Øystein Ahlstrøm

Mona Aleksandersen

Nils Ivar Dolvik

Anna Vigdis Eggertsdottir

Wenche Farstad

Constanze Fintl

Bjørn Kåre Gjerde

Gjermund Gunnes

Frøydis Hardeng

Lisbeth Hektoen

Carl Fredrik Ihler

Silje-Kristin Jensen

Thora J. Jonasdottir

Madelaine Norström

Charles McLean Press

Kristian Prydz

Birgit Ranheim

Ellen Skancke

Eystein Skjerve

Hege Kippenes Skogmo

Henning Sorum

Maria Stokstad

Arnfinn Sundsfjord

Anne Cathrine Whist

**Poland**

Malgorzata Brzóska

Henryka Dlugonska

Jacek Gajek

Magdalena Garncarz

**Portugal**

Luis Antunes

Madalena Vieira-Pinto

**Singapore**

Anbu Karuppannan

**Slovakia**

Peter Chrenek

Martina Klinkon

**South Africa**

Leith Meyer

**Spain**

Alicia Aranaz

Jose Ceron

Marina Sibila

**Sweden**

Pia Haubro Andersen

Eva Axnér

Kerstin Bergvall

Johan Bröjer

Agneta Egenvall

W. Freddy Fikse

Pia Funkquist

Ulrika Grönlund

Jenny Hadrevi

Jens Häggström

Anna Hillström

Odd Viking Höglund

Bodil Strom Holst

Désirée Jansson

Erika Karlstam

Clarence Kvart

Anne-Sofie Lagerstedt

Charles Ley

Ann Lindberg

Fernando Lopes Pinto

Ann Pettersson

Bengt Röken

Rolf Sporndly

Jonas Tallkvist

Anna Tidholm

Katarina Varjonen

Per Wallgren

Gunilla Westermark

Cecilia Wolff

**Switzerland**

Daniela Gorgas

Peter Kook

Mariusz P. Kowalewski

Virgile Lecoultre

Tosso Leeb

Bart van den Borne

Reto Zanoni

**Turkey**

Cengiz Gokbulut

**United Kingdom**

Elsa Beltran

Mark Bowen

Marnie Brennan

Leo Calvo-Bado

Sally Cutler

Pierpaolo Di Giminiani

Sue Dyson

Guillaume Fournié

Edward Hall

Mark Holmes

Jordi Lopez-Alvarez

Paul MacFarlane

Catherine McGowan

Jasmin Paris

Susan Paterson

Maxime Ratinier

Johnny Roughan

Keith Thoday

Nicola Williams

Ruth Zadoks

**United States of America**

Mohammed Altikriti

Frank Andrews

Fred Angulo

Matthew Binns

Douglas DeBoer

Mark Drew

Bernd Driessen

Jitender Dubey

Susan Eades

James Evermann

Daniel Feeney

James Ferguson

Philip Fox

Brandon Fraser

Chris Gardiner

H. Carl Gelhaus

Rita Hanel

Darryl Heard

Robert Henry

Karsten Hueffer

Katherine James

Michele Jay-Russell

Mark Jenkins

Shylo Johnson

James Jones

Anil Kadegowda

Michelle Kutzler

Donald Lay

Tomasz Leski

Jane Manfredi

T. G. Nagaraja

Stephen Nickerson

Jung Eun Park

Valerie Parker

Maria Pieters

Monica Santin-Duran

Geof Smith

Gary Splitter

Nathaniel Tablante

Kevin Washburn

Robert Weedon

